# Exploring long-term electrification pathway dynamics: a case study of Ethiopia

**DOI:** 10.1007/s43937-023-00014-4

**Published:** 2023-01-25

**Authors:** Andreas Sahlberg, Will Usher, Ioannis Pappis, Oliver Broad, Fitsum Salehu Kebede, Tewodros Walle

**Affiliations:** 1 Department of Energy Technology, Division of Energy Systems, KTH Royal Institute of Technology, Brinellvagen 68, 10044 Stockholm, Sweden; 2 Center for Renewable Energy, Addis Ababa Institute of Technology, Addis Ababa University, P.O. Box 385, Addis Ababa, Ethiopia; 3 UCL Energy Institute, University College London, Central House, 14 Upper Woburn Place, London, WC1H0NN UK; 4 Laboratoire IREENA, Nantes Université, 37 Boulevard de l’Université, Nantes 44602 Saint-Nazaire, France

## Abstract

The Open Source Spatial Electrification Tool (OnSSET) is extended to provide a long-term geospatial electrification analysis of Ethiopia, focusing on the role of grid- and off-grid technologies to increase residential electricity access under different scenarios. Furthermore, the model explores issues of compatibility between the electricity supply technologies over time. Six potential scenarios towards universal access to electricity in the country are examined based on three pathways; the *Ambition pathway* sees high demand growth and universal access achieved by 2025, the *Slow Down* pathway follows a lower demand growth with a slower electrification rate and with a higher share of off-grid technologies, and the *Big Business* pathway prioritizes grid electricity first for the industrial sector, leading to slower residential electrification. The results show a large focus on grid extension and stand-alone PV deployment for least-cost electrification in case of low grid-generation costs and uninhibited grid expansion. However, in case of a slower grid rollout rate and high demand growth, a more dynamic evolution of the supply system is seen, where mini-grids play an important role in transitional electrification. Similarly, in the case where grid electricity generation comes at a higher cost, mini-grids prove to be cost-competitive with the centralized grid in many areas. Finally, we also show that transitional mini-grids, which are later incorporated into the centralized grid, risk increasing the investments significantly during the periods when these are integrated and mini-grid standards are not successfully implemented. In all cases, existing barriers to decentralized technologies must be removed to ensure off-grid technologies are deployed and potentially integrated with the centralized grid as needed.

## Introduction

Access to electricity is an integral part of Sustainable Development Goal 7 (SDG 7), which is to *ensure access to affordable, reliable, sustainable and modern energy for all* by 2030 [1]. It has been shown that access to electricity shows positive synergies with ~ 85% of the sub-targets of all the SDGs [2]. Still, 759 million people globally lacked access to electricity as of 2018. Furthermore, 660 million people could remain without electricity by 2030 at the current rate of progress of electrification, falling short of achieving SDG 7 [3]. The situation has been further hampered by the Covid-19 pandemic, disrupting progress in many countries and making electricity unaffordable to an estimated 30 million people who had access to electricity before the pandemic [3].

In countries where universal access to electricity is yet to be reached, it has been shown that this can be achieved at the lowest cost through a combination of centralized grid and off-grid (mini-grid and stand-alone) technologies [4–6]. The question of *which* of these technology types and energy resources are to be used *where* is highly affected by spatially explicit information [4]. The cost of extending the centralized grid network to a new settlement depends on where the settlement is located to the existing grid network, because of the distance to be covered by a new grid line and the terrain that that line has to cross. Similarly, new run-of-river hydropower mini-grids can only be developed where there is a river nearby. In the existing literature, geospatial electrification models have therefore been used to examine the least-cost combination of grid-connected and off-grid technologies in both national studies [7–18] and regional studies for all of sub-Saharan Africa (SSA) [4–6, 19, 20]. These models draw on spatial information regarding energy resource availability, population, demand and distance to infrastructure in each settlement of the study area combined with techno-economic parameters to identify the least-cost technology in each location.

Previous geospatial electrification studies have shown that the choice of technology for least-cost electrification in each location is strongly linked to the level of demand in that location. These studies show that stand-alone technologies generally are the least-cost solutions at lower electricity demand levels, whereas higher demand levels favor mini-grids and extension of the centralized grid network instead [4, 20]. The Multi-Tier Framework (MTF) for electricity access defines five Tiers of electricity access, where Tier 1 provides the most basic services and implies a small consumption of electricity, and higher Tiers provide increasingly more services and at correspondingly larger consumption levels. The MTF also suggests stand-alone technologies as the typical supply technology for low levels of electricity access, with grid and mini-grids being best suited for Tier 3–5. Apart from the cost perspective seen in geospatial electrification studies, this framework also takes into consideration several other attributes, such as availability, reliability and quality of supply for the different Tiers [21].

Most of the previous geospatial electrification studies in the scientific literature have a relatively short time span, focusing on achieving SDG 7 by 2030, but do not provide further insights into the evolution of the system as demand increases beyond that timeline. Both the regional and national studies mentioned above examine scenarios to provide universal electrification either for the existing population in the study area (e.g. [5, 11, 22]), or for the projected population until 2030 (e.g. [15, 23, 24]). Pappis et al. provide a notable exception, extending the timeline until 2070 to examine three long-term pathways for the power sector in Ethiopia. They used the Open Source Spatial Electrification Tool (OnSSET) geospatial electrification tool coupled with the Open Source Energy Modelling System (OSeMOSYS) tool for optimizing grid-connected power supply, to examine three electrification pathways for the country [25]. Dalla longa et al. modelled electrification solutions in Ethiopia under a longer timeline, between 2020 and 2050. They used geospatial analysis to identify the shares of the population that could be served by grid extension, off-grid technologies or a combination of both in each 10-year interval until 2050. Thereafter they used the TIAM-ECN model to optimize the mix of centralized power plants as well as the combination of off-grid technologies. While the TIAM-ECN model in itself is not geospatial, it made use of the information from the initial geospatial analysis [26].

The deployment of off-grid technologies for electrification is a dynamic and complex process that varies over time. The results of Pappis et al. suggest that a technology change could occur in many locations in Ethiopia throughout the timeline they considered. A common theme across the three pathways they studied is a transition towards mini-grids and centralized grid extension as demand grows over time. This was largely a result of population growth, as well as socio-economic development leading to higher residential per capita consumption [25]. Experience from other countries shows that the arrival of the centralized grid network to locations previously served by mini-grids can be handled in different ways. Examples from Cambodia, Sri Lanka and Indonesia highlight these options and the challenges that they might face [27]. In the worst case, the existing mini-grid assets are abandoned as the centralized grid arrives. More successful examples are those where the distribution network keeps operating, but the mini-grid transforms so that all its electricity is supplied by the centralized grid (the mini-grid becomes a Small Power Distributor—SPD). In some cases, both the distribution and generation assets of the existing mini-grid are kept in operation. This can happen either through the interconnection of the mini-grid with the centralized grid, or by the mini-grid operating in parallel with the centralized grid [27].

In this study, we expand on the previous literature by focusing on the system dynamics from a long-term perspective. In particular, we study the effects of an optimal vs. a limited rate of expansion of the centralized grid network. Furthermore, we examine the costs associated with the change of technologies in a settlement. Based on a review of the existing literature, this is the first time these issues have been explored in a long-term geospatial electrification study extending beyond the expected lifetime of most off-grid technologies. Pappis et al. explore three long-term electrification pathways with, among other things, different outlooks for grid expansion [25]. Usher et al. also present two additional pathways for Ethiopia using the same methodology [28]. However, they do not explore further how these pathways are affected if the grid conditions change. Furthermore, they assume perfect integration of technologies, i.e. that both mini-grid generation and distribution assets keep operating in parallel or are fully integrated if the centralized grid arrives. Dalla Longa et al. [26] does not provide a full geospatial analysis of how the system evolves and finds only a minimal expansion of the grid in areas where it is competing with off-grid technologies in their universal electrification scenario. To explore these long-term dynamics, we expand on three of the pathways for Ethiopia presented by Usher et al. [28] by incorporating scenarios with and without restrictions to centralized grid expansion as well as scenarios with sub-optimal integration of electrification technologies.

## Background

Increased access to electricity is an important issue in Ethiopia. With 59 million people lacking access in 2018, Ethiopia has the third-highest deficit in SSA measured in absolute numbers [29]. In that year, only 44% of the population in the country had access to at least basic electricity services, 33% through the centralized grid and 11% through off-grid solutions [30]. In light of this situation, the country launched its ambitious National Electrification Program II (NEP II) in 2019, aiming to achieve universal access to electricity in the country by 2025 [31]. A geospatial analysis revealed that the large majority of people live close to the existing grid, with 90% of Ethiopian households located within 10 km. In light of this analysis, the NEP II aims to first connect 65% of the population to the centralized grid by 2025, focusing on those residing closest to the existing network first. The remaining 35% would receive electricity from off-grid technologies in that year. Thereafter the grid should expand outwards to connect 96% of the population to the grid by 2030, leaving 4% still supplied by off-grid technologies [31].

Despite the ambitious targets to achieve universal access to electricity by 2025, the energy situation in the country may have been exacerbated due to recent events. Because of the economic implications of the Covid-19 pandemic, an estimated 5–10% of the population may no longer be able to afford essential electricity services. Furthermore, the effects of the pandemic could also affect electrification progress negatively because of the financial effect on utility finances [3], higher costs of electricity supply technologies and disrupted global supply chains [32]. Additionally, the conflict in northern Ethiopia and drought pose additional socio-economic challenges within the country, and the war in Ukraine increases fuel prices globally [33]. All of these challenges risk negatively affecting the possibility to reach the NEP II targets, potentially lowering electricity demand, affordability and the capabilities of the national utility.

Pre-dating NEP II, GIS-based analysis of electrification scenarios for Ethiopia in the existing scientific literature displays diverse approaches to achieve universal access in the country. Mentis et al. applied a geospatial electrification model to examine scenarios for universal electrification in Ethiopia by 2030 [13]. They found grid connection to be the least-cost option for the majority of the population, with the role of off-grid technologies varying depending on demand. In a low-demand scenario, where the rural population targets 50 kWh/capita/year (Tier 2), grid connection was the least-cost option for 89% of the population, despite the low rural demand. Stand-alone technologies were the least-cost solution for 11% of the population and mini-grids for less than 1%. In a high-demand scenario, where the rural population targets 150 kWh/capita/year (Tier 3), grid connection was the least-cost alternative for 93% of new connections. At this demand level the role off the off-grid technologies was reversed; mini-grids were the least-cost option for most of the remote population not served by the grid (6% of the total population) with only a small role remaining for stand-alone technologies (1% of the total population). [13]. Dalla Longa et al. on the other hand projected universal access to electricity in the country to be achieved by 2040. Their results displayed a larger focus on off-grid technologies, suggesting they had the potential to provide affordable electricity for up to 70% of the Ethiopian population by 2050. The majority of the off-grid energy supply in this case would be renewable [26]. Pappis et al. explored one scenario where the NEP II targets are achieved, but also two scenarios where electrification occurs at a slower pace. In the latter two, off-grid technologies play a more pronounced role. In the case of slow demand growth, where a large share of the population remains at low per-capita demand levels throughout the study, stand-alone technologies play an important role during the whole modelling period. In case of faster demand growth, mini-grids play an important role to serve high-demand areas before the national grid eventually arrives [25]. Altogether, these studies show that from a least-cost perspective, there is a significant space for off-grid technologies as a means for electrification in Ethiopia both in the short-term and in a longer perspective.

The importance of off-grid technologies as a means for intermediate electrification in many areas in Ethiopia before the grid arrives, as evident from the NEP II targets and previous studies, indicates the need for a framework supporting the transition between technologies. Ahmed et al. [34] reviewed the regulatory and governance framework for decentralized electrification in Ethiopia. They found significant progress in this area in recent years, with the country fulfilling 14 out of 18 design features that they evaluated in 2021, whereas in 2017 the country fulfilled only seven out of these. Still, several underlying barriers to the deployment of off-grid technologies were identified, including complex licensing and approval procedures for mini-grids, low tariffs and limited lending support. On a positive note, a set of standards have been developed to ensure mini-grids are compatible with the centralized grid. However, they found that some stakeholders in the country were not aware of these [34].

## Methods

### Least-cost geospatial electrification model

**Fig. 1:**
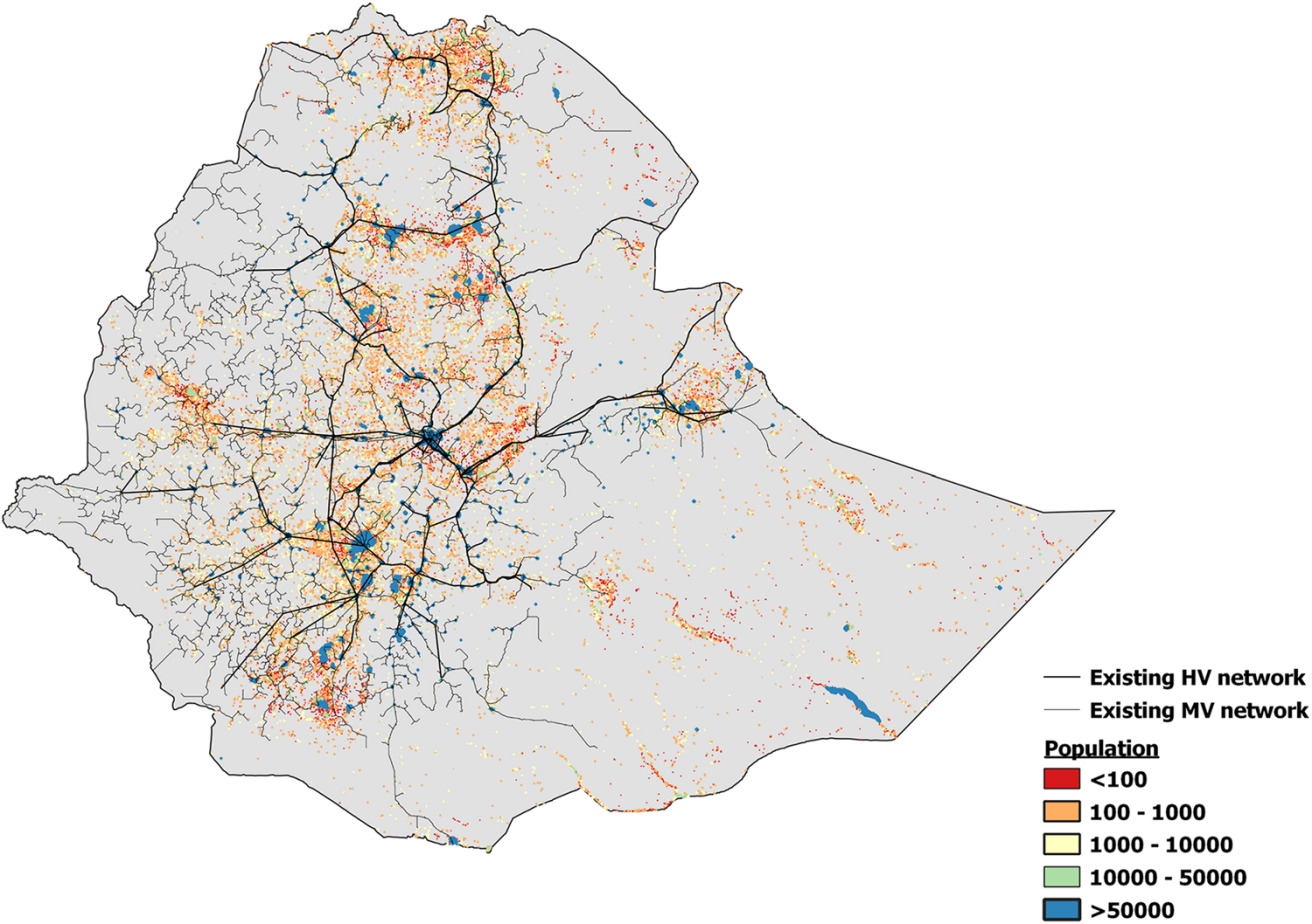
The current population, HV network and MV network in Ethiopia. The datasets sources are found in Appendix A. Note that MV lines displayed in Fig. 1 are from gridfinder.org. It can be seen that the majority of the populated areas are located near the HV and MV networks, apart from the southeastern parts of the country

In this study, the OnSSET tool is used to study long-term electrification pathways for Ethiopia until 2070. OnSSET draws on geospatial information regarding population, demand, energy resources and infrastructure as well as a set of demographic and techno-economic parameters to examine least-cost electrification scenarios [4]. In each settlement^1^ in the study area, the model identifies which technology can provide electricity at the lowest Levelized Cost of Electricity (LCOE) to meet the demand; connection to the centralized grid, mini-grid or stand-alone PV technologies. This process can be divided into four main steps:

1. **Extraction of GIS data**. In the first step of the analysis, geospatial information is identified for each settlement in the study area. This includes information on the settlement size and population, terrain, energy resource availability and distance to existing infrastructure. Figure 1 displays a selection of GIS datasets; population, High-Voltage (HV) network and Medium-Voltage (MV) network. The full list of GIS datasets used in the analysis is seen in Appendix A.
2. **Identification and projection of demand**. In the second step, the electricity demand is identified. In this paper, only residential electricity demand has been included. The annual demand (kWh/settlement/year) is calculated as the product of the projected number of households in the settlement in the year of interest and the expected demand per household. Furthermore, the peak load in each settlement is estimated by dividing the average annual load by an average-to-peak-load factor (for the full calculations, see [15]). The projected evolution of household demand levels over time in Ethiopia are further described in Sect. 3.2.
3. **Identification of least-cost off-grid electricity supply technology.** In the third step, the LCOE for each of the off-grid technologies is calculated, and the technology which can meet the demand at the lowest LCOE is identified as the least-cost off-grid technology in the settlement. For stand-alone, this includes the cost of the individual household systems. For mini-grids, this includes both the generation and storage system and the distribution network. The key techno-economic parameters used in this study are found in Appendix C. A full description of the cost evolution of both the on- and off-grid systems in Ethiopia is available in Pappis et al. [25].
4. **Grid-extension algorithm**. In the fourth step of the analysis, the model identifies settlements that can be supplied at a lower cost from connection to the grid compared to the least-cost off-grid technology. This includes the cost of extending new lines from the existing network to the settlement, constructing a distribution network within the settlement as well as the cost of electricity generation from the grid-connected power plants.

Recent versions of OnSSET ([15, 24]) include a time-step functionality following a myopic optimization process, where the least-cost technology deployment in the first time-step act as a starting point for electricity access in the next time-step, which acts as the starting point for the next time-step and so on [15]. In this study, the OnSSET model for Ethiopia is set up in six time steps from 2018 to 2070 (2018–2025, 2025–2030, 2030–2040, 2040–2050, 2050–2060 and 2060–2070). In each time step, the least-cost electrification technology is evaluated for each settlement. In settlements that are already electrified in the previous time-step, the model can choose to increase the capacity of the existing technology to meet the additional demand during the time-step,^2^ or to meet the demand using another technology. It is assumed that technological change can happen only in the direction of stand-alone to mini-grid to the centralized grid (e.g. a settlement where a mini-grid has been identified as the least-cost technology can only invest in new mini-grid capacity or connect to the centralized grid to meet the demand, it cannot use stand-alone technologies). The total residential electricity demand in a settlement is described by Eq. 1:

1 $${Etot}_{t}={POP}_{t}* {TIER}_{t}$$

where Etot_t_ is the electricity demand in the settlement (kWh/year) in time-step t, POP_t_ is the total population in the settlement in time-step t, and TIER_t_, is the per capita consumption Tier (kWh/capita/year) in the settlement in time-step t. There are five Tiers ranging from 1 to 5, where Tier 1 is lowest and Tier 5 is the highest. These Tiers are based on the Multi-Tier Framework for Measuring Energy Access [21], modified to reflect the specific appliances in the Ethiopian context.

Notably, in this study, the total study period considered is longer than the expected lifetime of the electricity supply technologies. The new demand to be supplied in a settlement in each time step is calculated from Eq. 2:

2 $${Enew}_{t}= {POP}_{t}* {TIER}_{t}- {POP}_{t-1}* {TIER}_{t-1}- Eexp$$

where Enew_t_ (kWh/year) is the new demand to be met in time-step t, POP_t-1_ is the population in the settlement in the previous time-step, TIER_t-1_ is the per capita consumption Tier (kWh/capita/year) in the settlement in the previous time-step, and Eexp (kWh/year) is the demand that was previously met by a technology whose technology lifetime expires during the time-step. Note that in a settlement that is unelectrified in the previous time-step, Enew_t_ = Etot_t_.

### Scenario analysis

Six (6) electrification scenarios for Ethiopia are developed, based on modified versions of three of the electricity development pathways seen in Usher et al. [28]. These three pathways describe different outlooks for Ethiopia in terms of socio-economic development, electricity demand growth of all sectors, electricity supply costs, cost of capital etc. By soft-linking OnSSET and OSeMOSYS, the cost of centralized grid electricity generation was also calculated from the optimal grid capacity expansion mix for each pathway [28]. In this study however, we focus only on residential electrification and residential electricity demand. As the projected residential demand of the currently unelectrified areas is significantly lower than the overall projected electricity demand in the country [28], the OSeMOSYS model is not updated in the present study. The three pathways included in this study are:

- The *Ambition* pathway represents an optimistic scenario, where Ethiopia achieves or even outperforms its goals. In this pathway, national electricity demand (all sectors included) grows rapidly and universal access to electricity is assumed to be achieved by 2025 as per the NEP II targets.
- In the *Big Business* pathway, the focus is placed on industrial consumers over residential electrification. National electricity demand grows rapidly due to economic growth, but residential electrification is assumed to follow past trends with universal access achieved by 2042.
- The *Slow Down* pathway represents a more pessimistic outlook, where national electricity demand grows more slowly and grid expansion is hampered in the short- and medium-term. Also in this scenario, electrification is assumed to follow past trends with universal access achieved by 2042.

For each of the three pathways, two grid-expansion scenarios are studied. According to NEP II, new grid connections in Ethiopia increased from 0.2 million in 2017 to 0.5 million in 2019. However, to achieve NEP II targets for grid connection, this number would need to ramp up to 1.7 million by 2025 [31]. In this paper, we modify the three pathways above to study the effects of the pace of roll-out of the grid. Two scenarios are modelled for each of the three pathways based on two different centralized grid options. In the *least-cost* scenarios, it is assumed that there is no limit to the pace of roll-out of the centralized grid, thus the grid will connect all settlements where it is the least-cost option. This can be considered the optimal electrification plan under each pathway. In the *restricted* scenarios, grid expansion is limited to a maximum of 500,000 households per year until 2030, based on the connections achieved in 2019, and ramp up afterwards. The key features of each scenario are summarized in Table 1.

**Table 1:** Key electrification supply features of the six scenarios examined in this study

Scenario	Universal electricity access year [28]	Demand growth [28]	Grid expansion	Grid generation cost [28]	Average annual population growth (%)	Average household size 2070 (people/household)	Discount rate (%)^a^ [28]
Ambition—least-cost	Universal access achieved by 2025	High	Unrestricted	Low (0.06–0.09 USD/kWh)	1.6	2.8	8.5
Ambition—restricted	Universal access achieved by 2025	High	Max 500,000 households/year until 2030, ramp up afterwards	Low (0.06–0.09 USD/kWh)	1.6	2.8	8.5
Big business—least-cost	Universal access achieved by 2042	Medium	Unrestricted	Low (0.06–0.09 USD/kWh)	1.6	3.0	8.5
Big business—restricted	Universal access achieved by 2042	Medium	Max 500,000 households/year until 2030, ramp up afterwards	Low (0.06–0.09 USD/kWh)	1.6	3.0	8.5
Slow down—least-cost	Universal access achieved by 2042	Low	Unrestricted	High (0.13–0.23 USD/kWh)	2.2	4.1	20
Slow down—restricted	Universal access achieved by 2042	Low	Max 500,000 households/year until 2030, ramp up afterwards	High (0.13–0.23 USD/kWh)	2.2	4.1	20

^a^The discount rate is used as a proxy for the cost of capital

The projected evolution of residential demand in each scenario relies on a combination of expert elicitation workshops and econometric projections as described in [28] to model the share of the population within each Tier of electrification level until 2070. During these expert elicitations, 18 stakeholders from the energy sector were invited to discuss possible futures for the energy system in Ethiopia. Attendees included members of public institutions and universities, private sector institutions, as well as donor organizations. The objective of the workshops was to discuss two key questions: (i) what do different energy supply pathways for Ethiopia look like and (ii) what are the current and future drivers of change in the Ethiopian energy system? To achieve this, discussions used a funnel approach to move from a wide description of the system, its actors, and its challenges, through to a common and categorized understanding of important thematic areas for its future development. These included energy technologies, policies and institutions, markets and finance, resources and environment, geographical context, and local capacity. These discussions provided the framework within which five qualitative pathways for the future development of the Ethiopian energy system were developed. Finally, each narrative pathway was linked to different input values for the econometric projections used to estimate the demand growth of each sector. In detail, the assumptions for growth in GDP, population, urbanization and household size drivers used in the projections were estimated for each pathway using the framework derived from the expert elicitation workshop. The residential demand for electricity was translated into the share of the population with different demand levels according to the modified MTF.

**Fig. 2:**
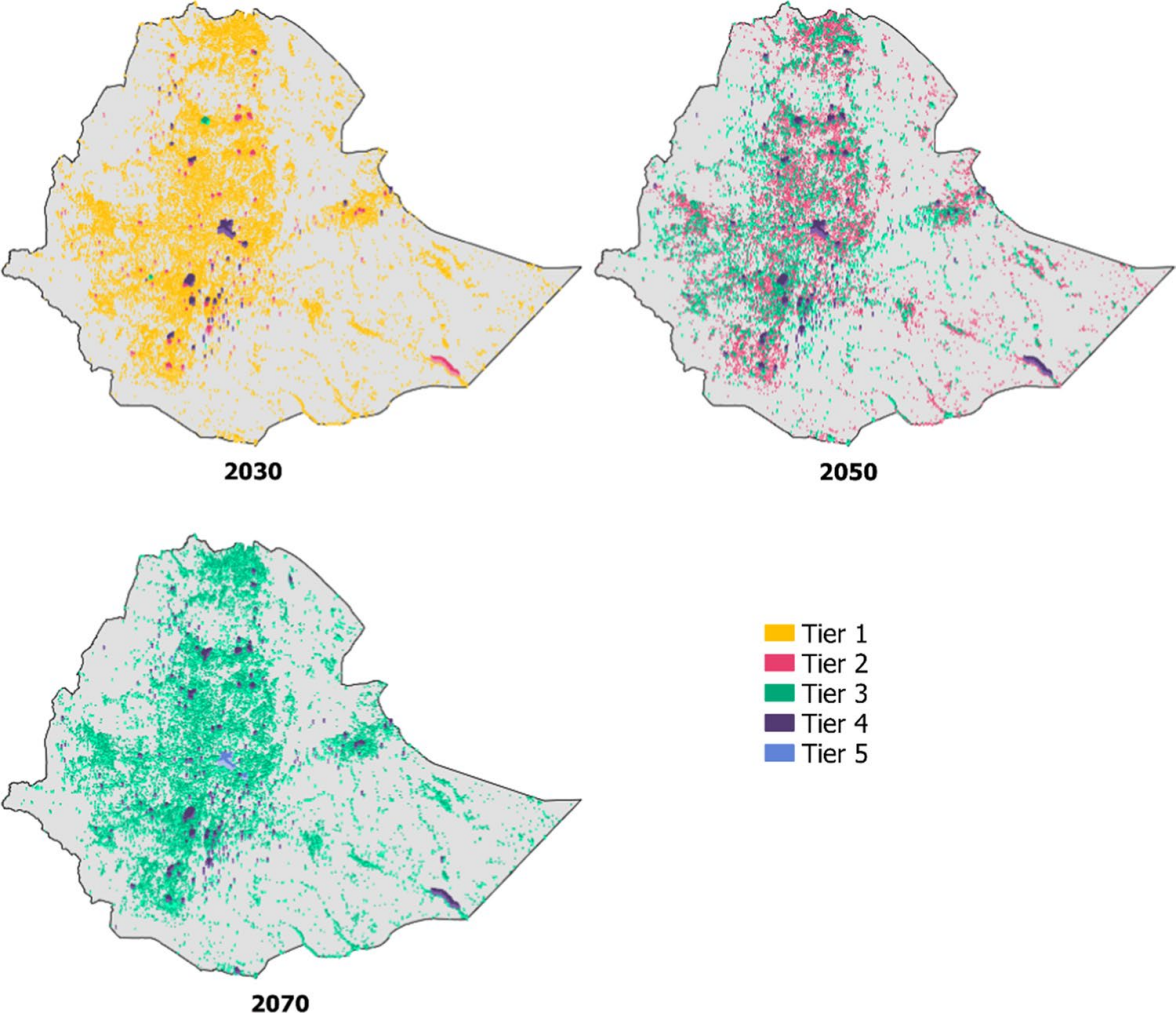
Distribution of demand Tiers in the Big Business scenarios in selected years (2030, 2050, 2070). The figure highlights the transition from Tier 1 to Tier 3 in the majority of smaller settlements, with an increasing number of larger settlements with Tier 4 or Tier 5 demand throughout the study

Spatially, it is assumed that the highest demand Tiers are found in the settlements with the largest populations, and the lowest Tiers in the settlements with the smallest populations. The spatial distribution of the demand Tiers for the Big Business scenarios is seen in Fig. 2. Similarly, the distribution for the Ambition and Slow Down scenarios is included in Appendix B.

This study further examines three levels of integration between the technology types (stand-alone, mini-grid and centralized grid) for the *restricted* scenarios. The *fully compatible* case assumes that technologies are fully compatible and keep operating as new technologies arrive to avoid any stranded investments. In this case, the existing capacity of generation and distribution technologies operates until the end of the technology's lifetime even if there is a technological change. When there is a technological change, the new technology in the settlement is sized to meet only the new demand occurring during the time step (only Enew_t_ is considered for the sizing of the new system). This represents a case where technologies can integrate fully from a technology, business and policy perspective. In the *semi-compatible* case, it is assumed that distribution networks of mini-grids remain in operation when the centralized grid arrives (mini-grids are converted to SPDs), but the mini-grid or stand-alone PV generation is replaced when there is a technology change. This means Etot_t_ is used to calculate the generation investments and Enew_t_ is used to calculate distribution investments in case of a technology change. In the *non-compatible* case, it is assumed that the different technology types are not compatible at all and that when a technology change occurs, the new technology replaces the previous technology completely and is sized to meet both the existing and new demand (Etot_t_ is used to calculate both generation and distribution investments in case of a technology change).

## Results and discussion

### Least-cost electrification scenarios

**Fig. 3:**
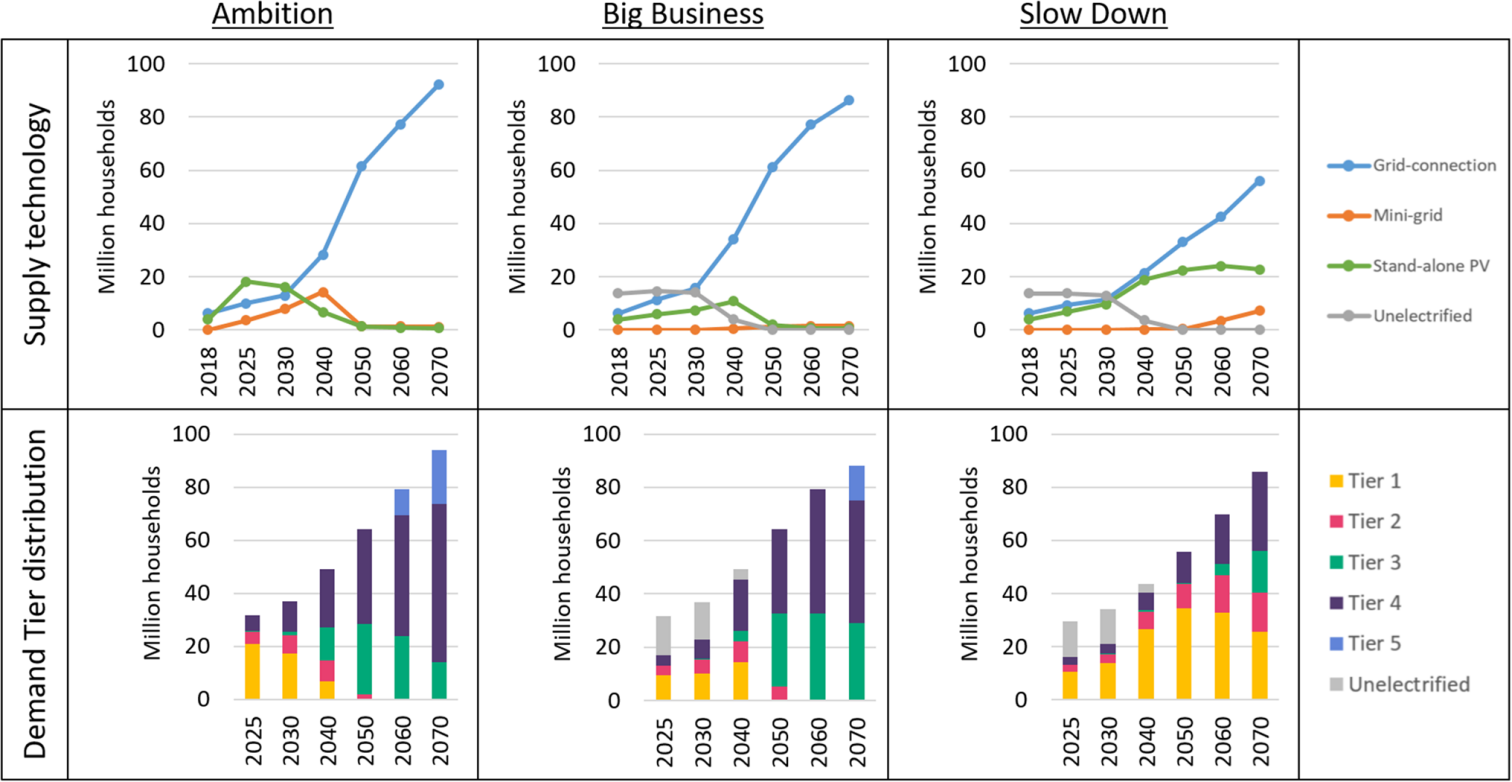
Top: Least-cost electrification technology for the three pathways in case of unrestricted grid expansion. Bottom: Demand Tiers in the three pathways. In the Big Business and Slow Down scenarios, not all demand is met until 2050, displayed as unelectrified in grey in the figure

In this section, the three energy transition pathways investigated in this study are presented. Figure 3 presents the evolution of the technology mix between 2018 and 2070 in the least-cost scenarios, to satisfy the different electricity demand Tiers the same period. These least-cost scenarios, assume the grid can expand unhampered by any limitations.

The Ambition scenario displays a clear link between the deployment of stand-alone PV technologies and the number of people with Tier 1 levels of demand. As demand levels increase and the population with Tier 1 demand decrease, the number of people supplied by stand-alone PV between 2025 and 2050 decreases as well. After 2050, the vast majority of the population has a demand level of Tier 3 or above, and stand-alone technologies provide the least-cost alternative for less than 2% of the population. Mini-grids supply a smaller share of the population throughout the modelling period. By 2030, these systems provide the least-cost alternative for 0.6 million people. These systems eventually grow to supply 3–4 million people from 2040 to 2070, the majority of which are PV mini-grids located more than 50 km from the current network. Given the relatively low grid generation cost in this scenario, and that 90% of the population lives within 10 km from the existing grid network, the grid can supply most of the population with demand levels of Tier 2 and above at the lowest cost.

The Big Business scenario follows a similar pattern as the Ambition scenario. Stand-alone PV systems follow the share of the population with Tier 1 levels of demand, and the rest of the population is supplied at the lowest cost by the centralized grid. However, demand levels increase at a lower rate, and overall electrification is slower compared to the Ambition scenario. Therefore, in 2025 and 2030, both stand-alone PV deployment and levels of grid extension are lower in this scenario. By 2040, when universal access to electricity is nearly achieved, the Big Business scenario shows a larger share of the population still supplied by stand-alone PV technologies compared to the Ambition scenario, explained by the slower demand growth. Similarly, the grid-connected population is lower by 2040 in the Big Business scenario. However, as in the Ambition scenario, by 2070 all of the population have Tier 3 – 5 levels of demand, with grid connection as the least-cost alternative for 98% of the population.

In terms of technology deployment, the Slow Down scenario displays the same correlation between the population with Tier 1 level of demand and stand-alone PV deployment as the previous two scenarios. With a slower demand growth than the previous two pathways, expansion of the grid is also slower and a larger share of the population is supplied by stand-alone PV technologies. However, in this pathway, there is also a larger deployment of mini-grids towards the end of the analysis. By 2070, 30 million people are served by mini-grids at the lowest cost. This is mainly due to the higher grid generation cost in this scenario, in combination with the falling costs of PV-based mini-grids, making mini-grids cost-competitive with the grid.

These three scenarios show that under an optimal least-cost development, where there are no restrictions to the pace of grid rollout, almost all but the most remote areas with high demand in Ethiopia are served at the lowest cost by grid-extension if grid electricity can be generated at a low cost (as seen in the Ambition and Big Business scenario). This can largely be attributed to the extensive grid network already in place in Ethiopia, allowing most of the settlements to be reached at a relatively low cost of grid extension given the short distances.

The investment costs in the three scenarios are strongly linked to the overall electricity demand (Table 2). In the Ambition scenario, where the demand is highest, total investments amount to 15.3 billion USD by 2030.^3^ This is similar to the 14.0 billion USD investment requirements identified for the NEP II [31]. Over the whole modelling period, the investments amount to 125 billion USD in the Ambition scenario, similar to the current GDP of the country (111 billion USD in 2021 [36]). In the Big Business scenario, given the slower electrification rate and demand growth, investments in both the short-term (until 2030) and over the whole modelling period are lower than in the Ambition scenario, at 8.3 and 96 billion USD respectively. In the Slow Down pathway, population growth is higher, with almost 40% more people projected to live in the country by 2070 in this scenario compared to the previous two. Still, investment requirements are significantly lower than the previous two scenarios; 5.5 billion USD until 2030 and 60 billion USD over the whole modelling period. This is explained by the significantly slower increase in demand levels in the Slow Down scenario.

**Table 2:** Undiscounted investment requirements in the least-cost electrification scenarios

Scenario	Investments 2018–2030 (billion USD)	Investments 2018–2070 (billion USD)
Ambition	15.3	125
Big Business	8.3	96
Slow Down	5.5	60
NEP II	14.0	–

### Restricted electrification scenarios

**Fig. 4:**
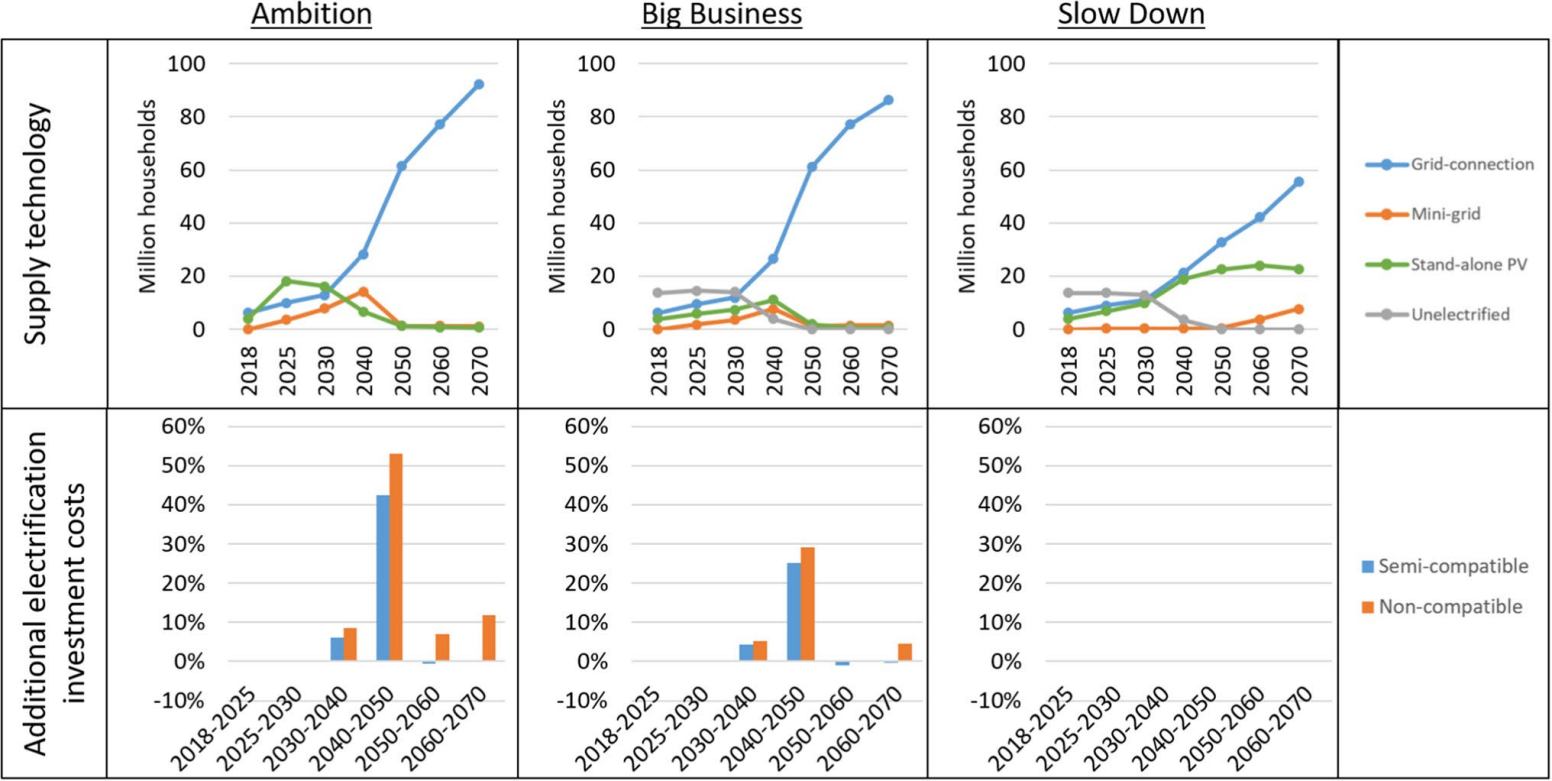
Top: Least-cost electrification technology for the three pathways in case grid expansion is limited. Bottom: Additional investment costs for settlements that are not grid-connected in the start year in case electricity supply technologies are semi-compatible or non-compatible compared to the fully compatible case

The three restricted scenarios, where grid connections are limited to current levels (maximum 500,000 households/year) and then ramp up, are seen to be more dynamic in terms of transition between the different electricity supply technologies than the unrestricted least-cost scenarios. Figure 4 displays the technology roll-out in these scenarios, as well as the difference in investment cost depending on the compatibility of electricity supply technologies for settlements that were not electrified at the start year of the analysis.

In the restricted Ambition scenario, the limitation to grid expansion is mainly countered by increased mini-grid deployment. This deployment increases investments compared to the least-cost Ambition scenario, to 22.4 billion USD by 2030, and 142 billion USD over the whole modelling period. In areas with high demand which cannot be reached by the grid, mini-grids are the least-cost option compared to stand-alone PV technologies in this scenario. Stand-alone PV technologies again peak early (by 2025), but are replaced by mini-grids and grid connections as demand levels increase. The deployment of mini-grids increases until 2040, after which the centralized grid ramp up to connect the vast majority of the population. By 2050, most mini-grids have been replaced. In the periods when the grid is replacing mini-grids, there are additional investment costs required where technologies are not fully compatible. This is most notable from 2040 to 2050, where additional investments for the *semi-compatible* and *non-compatible* cases are 42% and 53% higher respectively than the *fully compatible* case. This is the period where a significant number of mini-grids are replaced by the centralized grid network, as the ability to connect households to the grid is ramped up. Over the whole modelling period, total investments increased by 4.4% and 7.2% in the semi-compatible and non-compatible cases respectively.

In the restricted Big Business scenario, the limitation on grid expansion again increases investment costs compared to the least-cost scenario, to 11.5 billion USD by 2030, and 106 billion over the whole modelling period. This scenario follows a similar pattern of mini-grid expansion as the Ambition scenario. Mini-grids are increasingly deployed until 2040, after which they are replaced by connections to the centralized grid. Similarly, additional investments are seen to be required in the periods where mini-grids are replaced by a grid connection. These additional investments peak at 25–28% additional costs between 2040–2050, with total investments increasing by 3.8% and 4.9% for the semi-compatible and non-compatible cases respectively. Notably, these relative increases are lower than in the Ambition scenario. This is explained by a lower deployment of mini-grids, due to the lower demand growth (leading to more stand-alone PV instead) and a slower rate of electrification (leaving some potential mini-grid sites unelectrified before 2050) in the Big Business scenario.

In the restricted Slow Down scenario, investment costs are only marginally increased compared to the least-cost Slow Down scenario. Investments increase by 0.2 billion USD until 2030, and 1 billion USD over the whole modelling period. Given the slow demand growth in the Slow Down scenarios, connection to the centralized grid is only marginally higher in the unrestricted least-cost scenario than when the limitation on grid expansion is implemented in the restricted scenario. Mini-grids are the least-cost alternative for 1–2 million people until 2050 when grid expansion is restricted, then increases as in the least-cost scenario in the latter years. As there is virtually no transition from mini-grids towards grid connection in the Slow Down scenarios, there are no additional investment costs arising from semi-compatible or non-compatible electricity supply technologies.

These three scenarios highlight that if there are limitations to the pace of grid rollout, e.g. if the national utility’s performance is affected by the Covid-19 situation, mini-grids could play a more prominent role in increasing access to electricity. Mini-grids could then serve as an important stop-gap solution for high-demand areas until the centralized grid arrives. This stresses the importance of overcoming the remaining barriers in the governance and regulatory framework identified by Ahmed et al. [34]. Furthermore, the results of the restricted Ambition and Big Business scenarios highlight the significant additional costs that may incur if technologies are not compatible with each other. Over the whole modelling period, these effects on investment costs are lower than e.g. the effect of the different demand growths seen in the three pathways. However, if technologies are not compatible, significant investment increases are seen in the periods when large shares of the population move from mini-grids to centralized grid-connection. It is therefore crucial that the standards developed for grid integration are successfully implemented in new mini-grid projects if these mini-grids are to be integrated into the grid. The magnitude of these additional investments is directly linked to the magnitude of deployment of transitional mini-grids.

Both the least-cost scenarios and scenarios with limited expansion of the centralized grid highlight a couple of interesting aspects of stand-alone technologies in Ethiopia. First of all, they show that the role of stand-alone PV technologies for least-cost electrification is mainly driven by changes in demand levels. Across all six scenarios, there is a clear link between stand-alone PV deployment and the population with a Tier 1 level of demand. As people move towards higher demand levels towards the latter stages of each scenario, stand-alone PV is reduced throughout all of the scenarios. Secondly, stand-alone PV plays the largest role in the Slow Down scenarios, where demand growth is slow. In the Big Business and Ambition scenarios, where demand grows more rapidly, stand-alone PV plays a smaller role and is phased out earlier.

## Conclusions

In this study, we expand on previous long-term geospatial electrification studies for Ethiopia [25, 28] to study the role of grid- and off-grid technologies under scenarios with both optimal and limited rate of grid-expansion. Furthermore, extend the OnSSET tool to examine the costs associated with changes from transitional off-grid technologies as the centralized grid arrives in case these technologies are not fully compatible with each other.

Our results show that if grid electricity can be generated at a low cost, grid connection is the least-cost alternative for the majority of the population with high levels of electricity demand in Ethiopia. Similarly, stand-alone PV technologies are the least-cost alternative for the population with low levels of electricity demand. The split between these two depends largely on the growth in demand. However, if grid generation comes at a higher cost, or grid connections cannot accelerate quickly enough, there is a large space for mini-grids. Notably, in all six scenarios in this study, stand-alone technologies play a more prominent role compared to the NEP II targets.

Under medium or high demand growth with restricted grid expansion, as demand grows a dynamic system evolves where settlements switch between the different electricity supply technologies. This implies that the barriers to adapting the existing governance and regulatory framework must be overcome so that mini-grid technologies can act as intermediate solutions to provide electricity access in many high-demand locations before the grid arrives. Furthermore, the technical standards developed must be implemented to ensure additional costs due to stranded investments do not occur. The findings of this study can be relevant to many other countries where mini-grids are expected to play a significant role as an intermediate solution, whether that is in the short-, medium- or long term. Further studies could examine the effects of non-compatibility between electricity supply technologies in other geographies, especially in regions where the reach of the existing network is not as extensive.

It should be noted that the geospatial analysis in this study takes into account only residential electricity demand. Other sectors such as agriculture, social- and productive activities would add additional demands, which would affect the split between technologies. Further work should seek to also include those sectors. Finally, the code and data for the geospatial electrification analysis is published online^4^ and openly accessible to ensure reproducibility, replicability and reusability.

## Figures and Tables

**Fig. 5:**
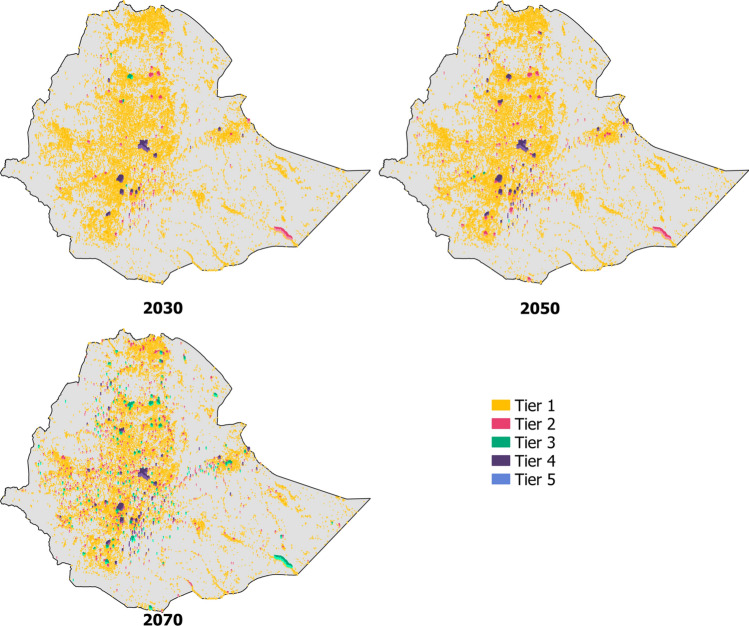
Distribution of demand Tiers in the Slow Down scenarios in selected years (2030, 2050, 2070). The highest tiers are seen in the settlements with the largest population. The figure shows many smaller settlements remaining with only Tier 1 demand, with an increasing number of larger settlements with Tier 2, Tier 3 or Tier 4 demand throughout the study

**Fig. 6:**
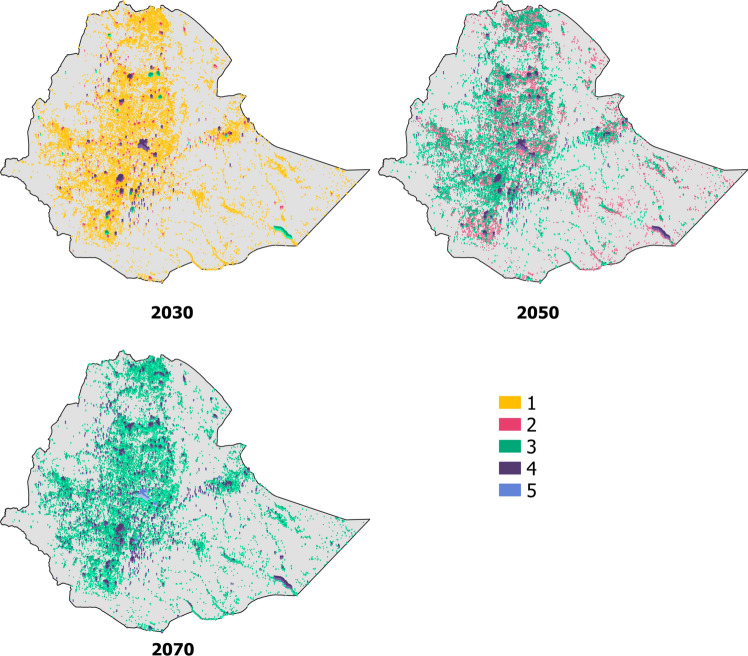
Distribution of demand Tiers in the Ambition scenario in selected years (2030, 2050, 2070). The highest tiers are seen in the settlements with the largest population. The figure highlights the transition from Tier 1 to Tier 3 in the majority of smaller settlements, with an increasing number of larger settlements with Tier 4 or Tier 5 demand throughout the study

## Data Availability

The datasets used during the current study are available in the GitHub repository: https://github.com/AndreasSahlberg/onsset-Ethiopia. This includes the processed GIS data for each settlement, ready to use in the OnSSET tool. The source GIS datasets, before processing, are listed in Appendix A.

## Appendix A—Geospatial datasets used in the analysis

**Table:**

Dataset	Type	Source
Population settlements	Shapefile	Khavari et al. [35]
Administrative boundaries	Shapefile	GADM [37]
Existing HV network	Shapefile	World Bank [38]
Existing MV network	Shapefile	Georeferenced from National Electrification Programme II [31]
Substations	Shapefile	Geofabrik / OpenStreetMap [39]
Roads	Shapefile	Geofabrik / OpenStreetMap [39]
Elevation	Raster	CGIAR-CSI [40]
Land cover	Raster	MODIS land cover [41]
Night-time lights	Raster	VIIRS [42]
GHI	Raster	Global Solar Atlas [43]
Wind speed	Raster	Global Wind Atlas [44]
Hydropower potential	Shapefile	KTH [45]
Travel time	Raster	Malaria Atlas Project [46]

## Appendix B—Demand Tier distributions

See Figs. 5, 6.

## Appendix C—Technology costs

See Tables 3, 4, 5

**Table 3:** Techno-economic parameters for off-grid technologies used in the OnSSET model

Technology	Overnight Investment Cost (USD/kW)						Efficiency (%)	Capacity factor (%)^a^	Operation and Maintenance cost (% of investment cost/year)
	2018–2025	2025–2030	2030–2040	2040–2050	2050—2060	2060–2070			
Diesel Mini-grid	721	721	721	721	721	721	33	70	10
Hydro Mini-grid	3000	3000	3000	3000	3000	3000	–	25	3
Solar PV Mini-grid	2829	2540	2252	1977	1703	1428	–	13–32	1.5
Wind Mini-grid	4356	4285	4213	4117	4021	3926	–	24–48	2
Solar PV Stand Alone (< 20 W)	9067	8143	7218	6338	5458	4577	–	13–32	2
Solar PV Stand Alone (20 – 50 W)	8487	7621	6756	5932	5108	4285	–	13–32	2
Solar PV Stand Alone (50—100 W)	6165	5537	4908	4310	3711	3113	–	13–32	2
Solar PV Stand Alone (100 – 1000 W)	4316	3876	3436	3017	2598	2179	–	13–32	2
Solar PV Stand Alone (> 1 kW)	6710	6026	5342	4690	4039	3387	–	13–32	2

The cost projections are developed by the authors based on a review of current costs, following the cost trends of the different technologies based on [46–48]

^a^Capacity factors for wind and solar technologies are calculated based on the annual resource availability in each settlement

**Table 4:** Centralized grid generation costs

Pathway	2018–2025	2025–2030	2030–2040	2040–2050	2050–2060	2060–2070
Grid generation cost (USD/kWh)						
Ambition	0.06	0.08	0.09	0.09	0.10	0.10
Big Business	0.06	0.07	0.08	0.08	0.09	0.08
Slow Down	0.13	0.17	0.19	0.19	0.20	0.23
Grid generation capacity investment cost (USD/kW)						
Ambition	3338	3354	2664	2222	2313	2447
Big business	2699	3196	2364	2285	2371	2242
Slow down	4436	4577	5624	2393	2778	3009

Derived from the OSeMOSYS model in [28]

**Table 5:** Transmission and distribution parameters, based on [4, 6, 15, 18, 23, 49–54]

Parameter	Value
HV line—69 kV	53,000 USD/km
MV line—33 kV	7000 USD/km
LV line—0.24 kV	4250 USD/km
Service transformer—50 kVA	4250 USD
Grid transmission and distribution losses	12%
Mini-grid distribution losses	5%

## Footnotes

1 A settlement in this study refers to anything from just a few households to a village or an entire city. For further information on the development of GIS settlements data for use in the OnSSET tool, see [35].

2 Note that additional demand is calculated based both on population growth and increased demand per household.

3 Note that all investments presented in the results are undicscounted. The discount rate presented in Table 1 is used to calculate the LCOE in order to compare the electricity supply technologies and choose the one that is the least-cost alternative.

4 https://github.com/AndreasSahlberg/onsset-Ethiopia

## Code availability

The OnSSET tool is open source, with the modified version of the tool used in the current study available in the GitHub repository: https://github.com/AndreasSahlberg/onsset-Ethiopia.
